# Impact of Smoking Reduction on Coronary Heart Disease Mortality Trends During 1981–2000 in England and Wales

**DOI:** 10.1186/1617-9625-1-17

**Published:** 2003-09-15

**Authors:** B Unal, JA Critchley, S Capewell

**Affiliations:** 1 Department of Public Health, University of Liverpool, UK; 2 Department of Public Health, Dokuz Eylul University School of Medicine, Izmir, Turkey

## Abstract

**Objective:**

To explore how much of the coronary heart disease (CHD) mortality fall in England and Wales can be attributed to changes in smoking prevalence.

**Methods:**

A previously validated cell-based IMPACT CHD mortality model was used to estimate the deaths prevented or postponed by changes in population smoking prevalence in England and Wales between 1981 and 2000. CHD mortality statistics and population trends in smoking were obtained from routine data sources.

**Results:**

In England and Wales between 1981 and 2000, smoking prevalence in adults aged 25–84 decreased from 43% to 28% in men and from 35% to 24% in women. In men, most of the decrease occurred in those aged over 55. Smoking prevalence changed little in older women. An estimated 29,460 deaths were prevented or postponed (DPP) by this population reduction in smoking prevalence. Most of this benefit was seen in men (86% of the DPPs versus 14% in women).

**Conclusion:**

Large declines in smoking prevalence accounted for 29,460 fewer CHD deaths in England and Wales in 2000 compared with 1981. This emphasises the importance of a national strategy with comprehensive tobacco control programmes to further reduce smoking.

## Introduction

Coronary heart disease (CHD) caused over 125,000 deaths in the UK in 2000. CHD mortality rates have been falling in most industrial countries since the 1970s [1]. Many authors attribute more of the CHD mortality fall to reductions in major risk factors such as smoking, cholesterol and blood pressure [2-4] rather than cardiological treatments [5,6]. It is suggested that between 20% [7] and 40% [8] of CHD deaths in men and women can be attributed to smoking in the UK.

Smokers demonstrate a two- to three-fold increase in the incidence of CHD compared with non-smokers[9], in men and women [10]. Smoking appears to increase CHD risk primarily through thrombosis (blood clotting). Some authors argue that smoking acts almost exclusively through thrombosis [11], while others suggest that smoking also promotes atherosclerosis [12]. If the main effect of smoking is thrombogenic rather than atherosclerotic, it would be plausible to expect that risk decline could occur rapidly on smoking cessation. The 1990 US Surgeon General's Report states that the risk is halved within 1–2 years and risk returns to that of a non-smoker after 15 years of abstinence [13].

Treating illness and disease caused by smoking is estimated to cost the NHS up to £1.7 billion every year in terms of general practitioner visits, prescriptions, treatment and operations [14]. To tackle the smoking problem and reduce smoking prevalence in the UK, the government published The White Paper on Tobacco in 1998 [15]. In this document, targets were set to reduce smoking among children from 13% to 9% or less, and among adults from 28% to 24% or less by 2010.

We therefore explored how much of the substantial CHD mortality fall in England and Wales between 1981–2000 could be attributed to reduction in smoking prevalence in the population.

## Methods

The cell-based IMPACT mortality model, previously validated in Scotland [16] and New Zealand [17], was further developed and refined to combine data for men and women, aged 25 to 84 in England and Wales describing: a) CHD patient numbers, b) uptake of specific medical and surgical treatments c) population trends in major cardiovascular risk factors (smoking, cholesterol, hypertension, obesity, diabetes, physical activity and deprivation) and d) effectiveness of specific cardiological treatments and risk factor reductions.

### Identification and assessment of relevant data

#### Population and patient information

Information on population, demographic changes, mortality and acute myocardial infarction incidence was based principally on routine health statistics from the Office for National Statistics [18,19] and the British Heart Foundation's Annual CHD Statistics [7].

#### Population risk factor trend data

Changes in the prevalence of measurable risk factors, including smoking, cholesterol, diabetes and blood pressure, were principally obtained from The British Regional Heart Study[20], the General Household Survey (GHS) [21], and the Health Survey for England [22]. Good data on smoking prevalence trends were easily available from successive General Household Surveys since 1974 [21,23]. The GHS is a continuous multipurpose survey of people living in private households, conducted by the Social Survey Division of the Office for National Statistics. A representative sample of all households is drawn from the postal address file. These households are then visited and data are collected on a wide range of matters for all residents aged 16 and over. In even-numbered years individuals are asked questions on smoking.

### The IMPACT Model

The current IMPACT Model aimed to include all medical and surgical treatments given in 1981 and 2000. The interventions considered in this study were those used in earlier versions of the IMPACT Model [16,17], along with primary angioplasty for myocardial infarction, statins in primary prevention, PG IIB/IIIA inhibitors for unstable angina, and spironolactone and beta blockers for heart failure (Appendix 1). Obesity, diabetes, physical activity and deprivation were the new cardiovascular risk factors included in the model.

The Microsoft Excel cell-based CHD mortality model has been described in detail elsewhere [16,17]. In brief, the number of CHD deaths expected in 2000 if mortality rates had not fallen since 1981 was calculated by indirect age standardisation using 1981 as a base year.

### Calculation of treatment effectiveness

The number of CHD deaths prevented or postponed in England and Wales in 1981, and again in 2000, were calculated for specific interventions, such as thrombolysis, coronary artery bypass grafting, aspirin and so on. Each specific mortality reduction was derived from the relative mortality reductions reported in published randomised controlled trials and meta-analyses. Survival benefit over a minimum time interval of one year was calculated for all treatments and all patient groups both in hospital and in the community[16] as in the example below:

#### Men aged 55–64 given aspirin for acute myocardial infarction

In the Antithrombotic Trialists' Collaboration meta-analysis, aspirin reduced relative mortality in men with acute myocardial infarction by 15% [24]. In England and Wales in 2000, 10,699 men aged 55–64 were eligible for aspirin treatment for acute myocardial infarction, and 95% received aspirin [25]. One year case fatality in men aged 55–64 admitted with an acute myocardial infarction was approximately 17% [26].

The deaths prevented or postponed for at least a year were therefore calculated as: *Patient numbers × treatment uptake × relative mortality reduction × one-year case fatality = 10,699 × 95% × 15% × 17% = 259 deaths prevented or postponed.*

Some uncertainty obviously surrounded each of the estimates. Therefore a sensitivity analysis was performed using analysis of extremes method [27] whereby the maximum and minimum feasible values were fed into the model. For therapeutic effectiveness, 95% confidence intervals for relative risks from meta-analyses were used as maximum and minimum estimates. Patient numbers were assumed to be reasonably accurate (no more than 10% higher or lower than our "best" estimates), but treatment uptake was more uncertain (values 50% higher or lower). By multiplying through, the resulting product then generated maximum and minimum estimates for deaths prevented or postponed.

#### Efficacy data

Data on efficacy of interventions and risk factor changes were obtained from published randomised controlled trials, meta-analyses and population studies. Each β coefficient (quantifying the fall in CHD mortality rate attributable to the change in a specific risk factor prevalence) in the existing IMPACT Model was reviewed and updated to include the latest pooled β coefficients published from MONICA and elsewhere[1].

There were a range of different coefficients or relative risks describing the relationship between the risk factors and coronary heart disease mortality (Appendix 2). Regression (β) coefficients identified for smoking ranged between 0.4 [28] and 0.73 [1] implying a 0.4%–0.73% fall in CHD mortality for every 1% fall in smoking. The best estimate for smoking 0.51 was taken from the MONICA study in Iceland [4]. The β coefficients for smoking, cholesterol and blood pressure were reduced among groups aged >65 years to reflect good epidemiological evidence suggesting that relative risk is attenuated by age [29,30].

### Risk factor trends and mortality benefits

The CHD mortality reduction attributable to declines in specific risk factors was principally based on a regression method. This used the mean β coefficients for smoking, cholesterol and blood pressure derived from eight pooled MONICA cohort studies in Finland, Iceland and Australasia [2-4,28].

The deaths prevented or postponed between 1981 and 2000 were calculated as: *CHD deaths in that age group in 1981 × risk factor decline × β coefficient.*

In England and Wales, in men aged 55–64 there were 18,255 CHD deaths in 1981; smoking prevalence fell from 43% to 24% between 1981–2000, an absolute reduction of 19%, and a relative reduction of **44%**. Best estimate of β coefficient for smoking was **0.51**.

Thus, **18,255 × 44% × 0.51 = 4,096 deaths **were prevented or postponed due to a fall in smoking prevalence in men aged 55–64 between 1981 and 2000.

This calculation was then repeated a) for men and women in each age group, b) for each risk factor, and c) for maximum and minimum values in each group.

### Validation of IMPACT Model

The model estimate for the total deaths prevented or postponed by all treatments plus all risk factor changes was then compared with the observed falls in mortality for men and women in specific age groups. There was generally good agreement between estimated and observed falls for men and women in each age group. Overall, the model explained 91% of the CHD mortality fall.

## Results

Between 1981 and 2000, CHD mortality rates fell by 55% in England and Wales, with 68,230 fewer CHD deaths than expected in men and women aged 25 to 84.

Some 42% of this fall was attributed to treatments in individuals and 58% to population risk factor changes [31]. Coronary heart disease treatments produced a best estimate of 25,955 fewer deaths (minimum estimate 13,390, maximum 39,295). Declines in the major cardiovascular risk factors together produced a best estimate of 36,110 fewer deaths (minimum estimate 18,950, maximum 43,820).

The majority of this fall was attributable to reductions in population smoking prevalence (43%), cholesterol (13%), and blood pressure (10%) [31].

**Figure 1:**
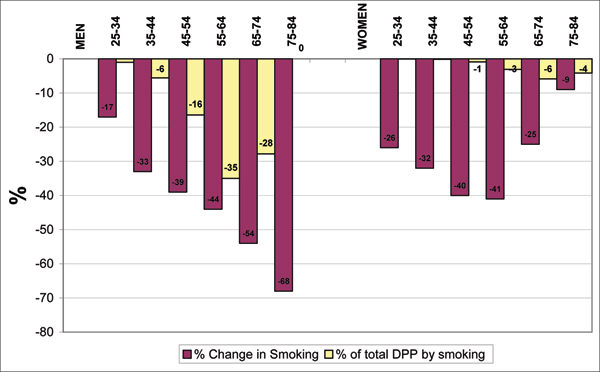
**Percentage change in smoking prevalence and % distribution of deaths prevented or postponed (DPP) attributed to smoking decline by sex and age group**.

Smoking prevalence decreased from 43% to 28% in men and from 35% to 24% in women between 1981–2000. In men, most of the decrease was in those aged over 55. Smoking prevalence changed little in older women (Table 1). There were an estimated 29,460 (13,885–43,230) DPP by this population reduction in smoking prevalence. Most of this impact was in men (86% of the DPPs were in men and 14% in women) (Figure 1).

**Table 1:** Change in CHD deaths, smoking prevalence in men and women, England and Wales, 1981–2000

**Sex and Age Groups**	**CHD Mortality Rates (per 10000)**			**Smoking Prevalence (%)**			**Deaths Prevented/Postponed**
	**1981**	**2000**	**Relative Change %**	**1981**	**2000**	**Relative Change %**	**Number (min-max)**
**Men**							
25–34	6.5	2.4	**-63**	47	39	**-17**	**24**
35–44	50.8	18.7	**-63**	46	31	**-33**	**302**
45–54	249.4	89.3	**-64**	46	28	**-39**	**1,632**
55–64	680.6	282.4	**-59**	43	24	**-44**	**4,840**
65–74	1562.1	807.2	**-48**	35	16	**-54**	**10,303**
75–84	2926.8	1896.9	**-35**	34	11	**-68**	**8,187**
**Total**	**556.0**	**213.8**	**-62**	**43**	**28**	**-36**	**25,289 (11,523–35,469)**

**Women**							
25–34	1.3	0.6	**-54**	43	32	**-26**	**6**
35–44	8.5	4.5	**-47**	41	28	**-32**	**40**
45–54	47.4	18.7	**-61**	42	25	**-40**	**260**
55–64	196.4	78.4	**-59**	39	23	**-41**	**912**
65–74	659.1	335.2	**-49**	24	18	**-25**	**1,727**
75–84	1726.7	1053.3	**-39**	11	10	**-9**	**1,227**
**Total**	**315.6**	**173.3**	**-45**	**35**	**24**	**-27**	**4,172 (2,365–7,760)**

**TOTAL**	**432**	**193.2**	**-55**	**39**	**26**	**-34**	**29,461 (13,888–43,229)**

## Discussion

Between 1981 and 2000 CHD mortality halved and smoking prevalence decreased by a third.

This decline in smoking prevalence explained approximately 43% of the total CHD mortality fall. Substantial declines in smoking prevalence were seen in men, and also women between 1981 and 2000. The underlying reasons are complex and may reflect both social and physiological factors [32]. Smoking peaked in the 1950s and 1960s, and fell steadily in the 1970s [33]. In England and Wales this decline in smoking prevalence over more than two decades principally reflects a smoking cessation effect rather than a cohort effect. However, the fall in smoking prevalence appears to have begun to level off recently. The remarkably stable smoking trends suggest that cessation rates are no longer increasing. The prevalence of cigarette smoking in Britain is indeed in danger of increasing again over the next few years [34], simply because more children and young people are starting to smoke. Almost a quarter of Britain's 15-year-olds (21% of boys and 26% of girls) are regular smokers [23], despite the fact that it is illegal to sell cigarettes to children under age 16.

An adverse effect of smoking on life expectancy has been reported from many population surveys [35-37]. Using a computer simulation model, Tsevat et al estimated that population life expectancy would increase 0.8 year by smoking cessation in 35-year old men [38]. Substantial benefit from reduction in smoking would be expected for cardiovascular disease, chronic obstructive pulmonary disease and lung cancer [38]. Recognising the major impact of smoking on CHD and cancer mortality, bigger reductions in smoking prevalence should therefore be targeted.

All modelling studies such ours have limitations. They are particularly dependent on the quality and extent of data available on CHD risk factor trends and treatment uptakes. However, smoking data from General Household Survey are good. Due to the many limitations in the data, extensive sensitivity analyses were performed to take into account the possible uncertainties in our estimates. These did not greatly alter the relative contribution of each risk factor decline or treatment category.

Lag times between changes in risk factors and changes in disease differ among diseases. For many carcinogens, the delay between exposure to a carcinogen and overt disease may be 20–30 years whereas for cardiovascular disease lag times are much shorter [39]. For example on quitting smoking or reducing cholesterol, most of the risk reduction occurs within about 5 years [19,29]. Lag times may therefore not be very important when considering a 20-year time period as in this CHD mortality model.

In this study, a regression coefficient of 0.51, estimated from MONICA studies, was used to quantify the relationship between population changes in smoking prevalence and the consequent change in population mortality rates from CHD. However, the influence of smoking on CHD mortality may be even greater. The results from prospective observational studies conducted in populations with a decreasing smoking prevalence may be biased by the misclassification of study subjects during the follow-up as a result of smoking cessation. This results in an underestimation of the risk of CHD caused by smoking [40]. For instance, in a study from Finland, the association between baseline smoking status and CHD weakened markedly when the follow-up time was extended. The risk ratio of cumulative CHD mortality associated with smoking was 6.96 in the first 2-year follow-up and it decreased gradually to 2.06 at the 20-year follow-up [40].

Using similar methodology, smoking accounted for 36% of CHD deaths prevented or postponed in Scotland between 1975–1994 [16] and 30% in New Zealand between 1982–1993 [17]. Our findings were broadly consistent with studies from the US [41] and Europe [42]. This emphasises the importance of a national strategy to further reduce smoking with comprehensive tobacco control programmes.

Many countries started tobacco control programmes in the 1970s and have had considerable success in reducing smoking rates. Norway, Finland and Iceland all introduced advertising bans back in the 1970s, which were followed by substantial reductions in smoking rates or tobacco consumption [43,44]. In 1998, the UK Government published The White Paper on Tobacco [15], which aimed to reduce smoking prevalence from 28% in year 2000 to 24% by 2010 in the UK [45]. Despite some levelling off, the UK target may be achieved simply on the basis of current trends. In the US, smoking prevalence among adults was 24% in 2000 and the corresponding target is to reduce it to 12% by 2010 [46]. The US target appears difficult to achieve, whereas the UK target is not challenging enough.

## Appendix 1. Medical and surgical treatments included in the model: data sources for treatment uptake levels and relative risk reductions

**Table:** 

**TREATMENTS**	**Source (year)**	**Treatment Uptake in 2000 (average)**	**Source (year)**	**Relative Risk Reduction**
**Acute myocardial infarction**				
Community cardiopulmonary resuscitation	UKHAS-Norris, 1998[1]	46%	BRESUS-Tunstall-Pedoe[2]	10%
Hospital cardiopulmonary resuscitation	UKHAS-Norris, 1998[1]	99%	BRESUS-Tunstall-Pedoe[2]	30% or 15% average
Thrombolysis alone or in Combination	UKHAS-Norris, 1998[1]	35%	Collins(1996)[3]	12%
Aspirin	UKHAS-Norris, 1998[1]	79%	Antithrombotic Trialists' Collaboration (2002)[4]	15%
Primary angioplasty	David Cunningham, Myocardial Infarction National Audit Project (MINAP) (2002) – personal communication	4%	Keeley (2002)[5]	30%
Beta blocker	UKHAS-Norris, 1998[1]	19%	Freemantle (1999)[6]	4%
ACE inhibitor	UKHAS-Norris, 1998[1]	19%	Latini (2000)[7]	7%
**Secondary Prevention in CHD Patients**				
Aspirin	Ryan(2001)[8]	61%	Antithrombotic Trialists' Collaboration (2002)[4]	15%
Beta blocker	EUROASPIRE II(2001) [9;9]	37%	Freemantle (1999)[6]	23%
ACE inhibitor	EUROASPIRE II(2001) [9;9]	21%	Flather (2000)[10]	23%
Statin	Ryan(2001)[8]	50%	Pignone (2000)[11]	29%
Warfarin	EUROASPIRE II(2001) [9;9]	4%	Lau (1992)[12]	15%
Rehabilitation	EUROASPIRE II(2001) [9;9]	30%	Taylor (2002)	27%
**Chronic Angina**				
CABG surgery	Society of Cardiothoracic Surgeons of Great Britain and Ireland[13]	100%	Yusuf (1994)[14]	39%
Angioplasty	British Cardiac Intervention Society(2002)[15]	100%	Keeley (2002)[5]	16%
Aspirin	Ryan(2001)[8]	50%	Antithrombotic Trialists' Collaboration (2002)[4]	15%
Statins	Ryan(2001)[8]	10%	Pignone (2000)[11]	29%
**Unstable Angina**				
Aspirin alone	Fox (2002)[16], Collinson (2000)[17]	30%	Antithrombotic Trialists' Collaboration (2002)[4]	15%
Aspirin & Heparin	Fox (2002)[16], Collinson (2000)[17]	60%	Antithrombotic Trialists' Collaboration (2002)[4]	15%
Platelet glycoproteinIIB/IIIA inhibitors	Fox (2002)[16], Collinson (2000)[17]	50%	Boersma(2002)[18]	9%
**Heart Failure in the Hospital**	(2002)	46%		
ACE inhibitor	Clealand (2002)[19]	58%	Flather (2000)[10]	26%
Beta blocker	Clealand (2002)[19]	28%	Shibata (2001)[20]	37%
Spironolactone	Clealand (2002)[19]	10%	Pitt (1999)[21]	30%
Aspirin	Clealand (2002)[19]	50%	Antithrombotic Trialists' Collaboration (2002)[4]	15%
Statins	Clealand (2002)[19]	32%	Pignone (2000)[11]	29%
**Heart Failure in the Community**				
ACE inhibitor	Ellis (2001)[22]	68%	Flather (2000)[10]	26%
Beta blocker	Cleland (2002)[19]	17%	Shibata (2001)[20]	37%
Spironolactone	Cleland (2002)[19]	12%	Pitt (1999)[21]	41%
Aspirin	Ellis (2001) [22]	38%	Antiplatelet Trialists' Collaboration (1994)[23]	15%
Statin	Cleland (2002)[19]	43%	Pignone[11]	29%
**Hypertension**	Health Survey for England 1998(2001)[24]	59%	Collins (1990)[25]	11%
**Statins for primary Prevention**	Packham (2000)[26]	3%	Pignone[11]	29%

## Appendix 2. Methodological Issues: β coefficients and interactions between risk factors and treatments

There are a range of different coefficients or relative risks (RR) describing the relationship between each separate risk factors and CHD mortality. These vary somewhat in magnitude (see tables below).

**Table:** Estimated β coefficients from multiple regression analyses for the relationship between changes in population mean risk factors and changes in coronary heart disease mortality (men under 65 only)

	**Estimated β Coefficients**		
**Study**	**Smoking**	**Cholesterol**	**Blood Pressure**

**Our 'best' estimates**	**0.51**	**2.00**	**1.67**

MONICA, 2000 [27]	0.73	1.31	0.53

Vartiainen *et al. *1994 [28]	0.70	2.00	1.67

Sigfusson 1991 [29]	0.51	2.22	1.06

Dobson *et al. *1996 [30]	0.40	1.15	1.26

Collins/MacMahon, 1990 [25;31]	-	-	2.08

Seven Countries [32;33]	-	2.10	2.09

Law *et al. *1994 [34]	-	2.46	-

Vartiainen et al [28] and Sigfusson et al [29] are individual populations (Finland and Iceland respectively) from the MONICA study. Dobson et al 1996 [30] estimates are based on a subset of data from the MONICA study. Hence these estimates are not independent of each other. The major outcome in the MONICA 2000 [27] study was coronary event rate, as opposed to CHD mortality from the other MONICA studies.

The MONICA coefficients could be considered the most appropriate, being the study to consider the impact of changes in risk factors on changes in CHD mortality at a ***population ***level. However, the MONICA coefficients have been repeatedly criticised for 'ecological bias' and may underestimate the relationship between changes in risk factors and population trends in CHD mortality. This is because:

1) Those who do not respond to risk factor surveys may be at higher risk than attendees, and a decreasing response rate to MONICA surveys was observed over the course of the study [27].

2) The major outcome from the MONICA study was all coronary events, not just CHD mortality, which may be expected to slightly dilute the β coefficients obtained.

3) MONICA coefficients do not account for possible regression dilution bias; adjusted coefficients may be as much as 60% higher [34].

4) The principal MONICA estimates made no allowance for a possible lag time between changes in the risk factor levels and changes in population CHD mortality [27].

The MONICA coefficients for cholesterol and diastolic blood pressure are generally lower than from other sources [32,33], even constituent MONICA populations [28-30]. The MONICA coefficients have thus been used in our model as minimum estimates using the data for males only. In many cases, the number of events among females was too small to obtain reliable estimates, and the smoking coefficient appeared particularly anomalous. However, these global MONICA coefficients were mostly within the range of those estimated from individual populations in the MONICA study, with the possible exception of blood pressure.

Furthermore, these may be conservative, lacking the adjustment for regression dilution bias [35,36] recommended by some authors [34-36] but not all [37].

Coefficients derived from meta-analyses and the large cohort studies were regarded in our model as maximums. Maximum estimates were taken from Law et al for cholesterol [34], and Seven Countries for blood pressure [32,33], and best estimates were taken from the MONICA study in Iceland for blood pressure and smoking [29], and Finland for cholesterol [28]. The coefficients were reduced among older age groups to reflect good epidemiological evidence suggesting that relative risk is attenuated by age [34]. These 'maximum' coefficients may be overestimates being based on cohort analyses, which consider only the incremental effects of a risk factor on CHD mortality. These estimates are unlikely to be fully reversible when a population reduces its risk factor levels.

**Table:** 'Best' published values of relative risks for coronary heart disease mortality for obesity, diabetes, physical inactivity and deprivation.

	**Relative Risk**			
	**Obesity** (BMI>29 kg/m^2^)	**Diabetes**	**Physical activity**	**Deprivation**

**Men**	Stevens *et al*, 1998 [38], RRs ranged from 1.57 to 2.33^# ^by age groups.	Khaw *et al*, 2001 [39], RR = 4.24*(1.92–9.35)	Shaper *et al*, British Regional Heart study, 1991 [40] RR = 0.50** (0.2–0.8)	Davey-Smith *et al*. Renfrew and Paisley Study, 1998 [41], RR = 1.24(1.03–1.49)^+^

**Women**	Stevens *et al*, 1998 [38], RRs ranged from 1.00 to 2.24^# ^by age groups. Willett *et al*,1995 [42]RR = 3.5 6 (2.96–4.29)	Female RRs × 1.5 higher than male, (Members of the British Diabetic Association Study) [43].	Lee *et al*, Womens' Health Study, 2001 [44], RR = 0.55*** (0.37–0.82)	Davey-Smith *et al*, Renfrew and Paisley Study, 1998 [41], RR = 1.44 (1.15–1.80)^+^

# Adjusted for age, education, physical activity, alcohol consumption.

* Adjusted for age, serum cholesterol, systolic blood pressure, smoking, BMI, MI or stroke history.

** Adjusted for BMI, social class, smoking, total cholesterol, HDL cholesterol, FEV1, breathlessness and heart rate.

***Adjusted for age, treatment, smoking, alcohol, fat consumption, fibre, fruits and vegetables, use of hormones, postmenopausal status, parental history of MI at an early age.

+ Adjusted for age, blood pressure, cholesterol, BMI, FEV1 score, smoking, angina, ECG ischeamia, and bronchitis.

## Independence Issues

All these β coefficients and relative risks were obtained from multiple regression analyses; hence the interaction between the major risk factors should have been accounted for. However, these estimates may still overestimate because most models, of necessity, entered data into the model on only a limited range of risk factors. For the MONICA study, these are smoking (yes or no), systolic blood pressure, total cholesterol, and body mass index [27]. There are many other important risk factors for CHD, including diet (such as consumption of fish oils and anti-oxidants), physical activity, affluence, employment and education. Some may be highly correlated with the four risk factors measured. It is likely, therefore, that the calculated coefficients contain the effects of some of these changes at a population level, as well as those in the measured risk factor.

## REFERENCE LIST FOR APPENDICES

1. Norris RM. Fatality outside hospital from acute coronary events in three British health districts, 1994–5. United Kingdom Heart Attack Study Collaborative Group. BMJ 1998; 316:1065–70.

2. Tunstall-Pedoe H, Bailey L, Chamberlain DA, et al. Survey of 3765 cardiopulmonary resuscitations in British hospitals (the BRESUS Study): methods and overall results. BMJ 1992; 304:1347–51.

3. Collins R, MacMahon S, Flather M et al. Clinical effects of anticoagulant therapy in suspected acute myocardial infarction: systematic overview of randomised trials. BMJ 1996; 313:652–9.

4. Collaborative meta-analysis of randomised trials of antiplatelet therapy for prevention of death, myocardial infarction, and stroke in high risk patients. BMJ 2002; 324:71–86.

5. Keeley EC, Velez CA, O'Neill WW, Safian RD. Long-term clinical outcome and predictors of major adverse cardiac events after percutaneous interventions on saphenous vein grafts. J. Am. Coll. Cardiol. 2001; 38:659–65.

6. Freemantle N, Cleland J, Young P, Mason J, Harrison J. beta Blockade after myocardial infarction: systematic review and meta regression analysis. BMJ 1999; 318:1730–7.

7. Latini R, Maggioni AP, Flather M, Sleight P, Tognoni G. ACE inhibitor use in patients with myocardial infarction: Summary of evidence from clinical trials. Circulation 1995; 92:3132–7.

8. Ryan R, Majeed A. Prevalence of ischaemic heart disease and its management with statins and aspirin in general practice in England and Wales, 1994–98. Health Statistics Quarterly 2001; 12:34–9.

9. Lifestyle and risk factor management and use of drug therapies in coronary patients from 15 countries; principal results from EUROASPIRE II Euro Heart Survey Programme. Eur Heart J 2001; 22: 554–72.

10. Flather MD, Yusuf S, Kober L et al. Long-term ACE-inhibitor therapy in patients with heart failure or left-ventricular dysfunction: a systematic overview of data from individual patients. ACE-Inhibitor Myocardial Infarction Collaborative Group. Lancet 2000; 355:1575–81.

11. Pignone M, Phillips C, Mulrow C. Use of lipid lowering drugs for primary prevention of coronary heart disease: meta-analysis of randomised trials. BMJ 2000; 321:983–6.

12. Lau J, Antman EM, Jimenez-Silva J, Kupelnick B, Mosteller F, Chalmers TC. Cumulative meta-analysis of therapeutic trials for myocardial infarction. N. Engl. J. Med. 1992; 327:248–54.

13. Society of Cardiothoracic Surgeons of Great Britain and Ireland. Trends in Surgery for Ischaemic Heart Disease. . 17-2-0003.

14. Yusuf S, Zucker D, Peduzzi P et al. Effect of coronary artery bypass graft surgery on survival: overview of 10-year results from randomised trials by the Coronary Artery Bypass Graft Surgery Trialists Collaboration. Lancet 1994; 344:563–70.

15. de Belder, M. British Cardiac Intervention Society (BCIS) audit returns of interventional procedures 2000. . 17-2-2003.

16. Fox KA, Poole-Wilson PA, Henderson RA et al. Interventional versus conservative treatment for patients with unstable angina or non-ST-elevation myocardial infarction: the British Heart Foundation RITA 3 randomised trial. Randomized Intervention Trial of unstable Angina. Lancet 2002; 360:743–51.

17. Collinson J, Flather MD, Fox KA et al. Clinical outcomes, risk stratification and practice patterns of unstable angina and myocardial infarction without ST elevation: Prospective Registry of Acute Ischaemic Syndromes in the UK (PRAIS-UK). Eur. Heart J. 2000; 21:1450–7.

18. Boersma E, Harrington RA, Moliterno DJ et al. Platelet glycoprotein IIb/IIIa inhibitors in acute coronary syndromes: a meta-analysis of all major randomised clinical trials. Lancet 2002; 359: 189–98.

19. Cleland JG, Cohen-Solal A, Aguilar JC et al. Management of heart failure in primary care (the IMPROVEMENT of Heart Failure Programme): an international survey. Lancet 2002; 360:1631–9.

20. Shibata MC, Flather MD, Wang D. Systematic review of the impact of beta blockers on mortality and hospital admissions in heart failure. Eur. J. Heart Fail. 2001; 3:351–7.

21. Pitt B, Zannad F, Remme WJ et al. The effect of spironolactone on morbidity and mortality in patients with severe heart failure. Randomized Aldactone Evaluation Study Investigators. N. Engl. J. Med. 1999; 341:709–17.

22. Ellis C, Gnani S, Majeed A. Prevalence and management of heart failure in general practice in England and Wales, 1994–98. Health Statistics Quarterly 2001; 11:17–24.

23. Collaborative overview of randomised trials of antiplatelet therapy – I: Prevention of death, myocardial infarction, and stroke by prolonged anti-platelet therapy in various categories of patients. Antiplatelet Trialists' Collaboration [see comments] [published erratum appears in BMJ 1994 Jun 11; 308(6943):1540]. BMJ 1994; 308:81–106.

24. Primatesta P, Brookes M, Poulter NR. Improved hypertension management and control: results from the health survey for England 1998. Hypertension 2001; 38:827–32.

25. Collins R, Peto R, MacMahon S et al. Blood pressure, stroke, and coronary heart disease. Part 2, Short-term reductions in blood pressure: overview of randomised drug trials in their epidemiological context. Lancet 1990; 335:827–38.

26. Packham C, Pearson J, Robinson J, Gray D. Use of statins in general practices, 1996–8: cross sectional study. BMJ 2000; 320:1583–4.

27. Kuulasmaa K, Tunstall PH, Dobson AJ et al. Estimation of contribution of changes in classic risk factors to trends in coronary-event rates across the WHO MONICA Project. Lancet 2000; 355:675–87.

28. Vartiainen E, Puska P, Pekkanen J, Tuomilehto J, Jousilahti P. Changes in risk factors explain changes in mortality from ischaemic heart disease in Finland. BMJ 1994; 309:23–7.

29. Sigfusson N, Sigvaldson H, Steingrimsdottir L, et al. Decline in ischaemic heart disease in Iceland and change in risk factor levels. BMJ 1991; 302:1371–5.

30. Dobson A, Filipiak B, Kuulasmaa K et al. Relations of changes in coronary disease rates and changes in risk factor levels: methodological issues and a practical example. Am J Epidemiol 1996; 143:1025–34.

31. MacMahon S, Peto R, Cutler J et al. Blood pressure, stroke, and coronary heart disease. Part 1, Prolonged differences in blood pressure: prospective observational studies corrected for the regression dilution bias. Lancet 1990; 335:765–74.

32. Menotti A, Blackburn H, Kromhout D et al. Changes in population cholesterol levels and coronary heart disease deaths in seven countries. Eur Heart J 1997; 18:566–71.

33. van den Hoogen PC, Feskens EJ, Nagelkerke NJ, Menotti A, Nissinen A, Kromhout D. The relation between blood pressure and mortality due to coronary heart disease among men in different parts of the world. Seven Countries Study Research Group. N. Engl. J Med 2000; 342:1–8.

34. Law MR, Wald NJ, Thompson SG. By how much and how quickly does reduction in serum cholesterol concentration lower risk of ischaemic heart disease? BMJ 1994; 308:367–72.

35. Law M, Wald N, Wu T, Hackshaw A, Bailey A. Systematic underestimation of association between serum cholesterol concentration and ischaemic heart disease in observational studies: data from the BUPA study. BMJ 1994; 308:363–6.

36. Clarke R, Shipley M, Lewington S et al. Underestimation of risk associations due to regression dilution in long-term follow-up of prospective studies. Am. J. Epidemiol. 1999; 150:341–53.

37. Millo J. Hidden bias in observational study. BMJ 1994;308: 1038.

38. Stevens J, Cai J, Pamuk ER, Williamson DF, Thun MJ, Wood JL. The effect of age on the association between body-mass index and mortality. N. Engl. J. Med. 1998; 338:1–7.

39. Khaw KT, Wareham N, Luben R et al. Glycated haemoglobin, diabetes, and mortality in men in Norfolk cohort of european prospective investigation of cancer and nutrition (EPIC-Norfolk). BMJ 2001; 322:15–8.

40. Shaper AG, Wannamethee G. Physical activity and ischaemic heart disease in middle-aged British men. Br Heart J 1991; 66:384–94.

41. Smith GD, Hart C, Watt G, Hole D, Hawthorne V. Individual social class, area-based deprivation, cardiovascular disease risk factors, and mortality: the Renfrew and Paisley Study. J. Epidemiol. Community Health 1998; 52:399–405.

42. Willett WC, Manson JE, Stampfer MJ et al. Weight, weight change, and coronary heart disease in women. Risk within the "normal" weight range. JAMA 1995; 273:461–5.

43. Swerdlow AJ, Jones ME. Mortality during 25 years of follow-up of a cohort with diabetes. Int. J. Epidemiol. 1996; 25:1250–61.

44. Lee IM, Rexrode KM, Cook NR, Manson JE, Buring JE. Physical activity and coronary heart disease in women: is "no pain, no gain" passe? JAMA 2001; 285:1447–54.

## Competing interests

The authors declare that they have no competing interests.
